# Superior efficacy of Adalimumab in treating childhood refractory chronic uveitis when used as first biologic modifier drug: Adalimumab as starting anti-TNF-α therapy in childhood chronic uveitis

**DOI:** 10.1186/1546-0096-11-16

**Published:** 2013-04-15

**Authors:** Gabriele Simonini, Andrea Taddio, Marco Cattalini, Roberto Caputo, Cinzia de Libero, Fulvio Parentin, Ilaria Pagnini, Loredana Lepore, Rolando Cimaz

**Affiliations:** 1 Department of Paediatrics, Rheumatology Unit, Anna Meyer Children’s Hospital, University of Florence, Viale Pieraccini, Firenze 24 50139, Italy; 2 Institute for Maternal and Child Health - IRCCS “Burlo Garofolo” – Trieste, University of Trieste, Trieste, Italy; 3 Pediatric Clinic, University of Brescia, Brescia, Italy; 4 Department of Pediatrics, Ophthalmology Unit, Anna Meyer Children’s Hospital, University of Florence, Firenze, Italy; 5 Ophthalmology Unit, Institute for Maternal and Child Health - IRCCS “Burlo Garofolo” – Trieste, University of Trieste, Trieste, Italy

**Keywords:** Adalimumab, Infliximab, Children, Chronic uveitis

## Abstract

**Background:**

Nonetheless biologic modifier therapies are available treatment strategies for sight-threatening uveitis in children, the lack of evidence from head-to-head randomized controlled studies limits our understanding of timing of therapy when to commence therapy, which agent to choose and how long to continue treatment, and, in case of failure, if switching to another anti-TNF-α strategy might be eventually an option. Our aim was to compare the efficacy of Adalimumab when used as first anti-TNFα therapy *versus* Adalimumab used after the failure of a previous anti-TNFα (Infliximab) in an open-label, comparative, multi-center, cohort study of childhood chronic uveitis.

**Methods:**

26 patients (14 F, 12 M; median age: 8.6 years) with refractory, non-infectious active uveitis were enrolled. Due to the refractory course of uveitis to previous DMARD treatment, Group 1 received Adalimumab (24 mg/sq mt, every 2 weeks), as *first* anti-TNFα choice; Group 2 received Adalimumab, as *second* anti-TNFα drug, due to the loss of efficacy of Infliximab, administered after a period of at least 1 year. Both groups received Adalimumab for at least 1 year of treatment. Primary outcome was, once remission was achieved, the time to a first relapse.

**Results:**

14 children (10 with JIA, 3 with idiopathic uveitis, 1 with Behçet’s disease) were recruited in Group 1; 12 children (7 with JIA, 3 with idiopathic uveitis, 1 with early-onset sarcoidosis, 1 with Behçet’s disease) in Group 2. Group 2 showed a lower probability to steroid discontinuation during the first 12 months of treatment (Mantel-Cox χ^2^4.12, p<0.04). In long-term follow-up, Group 1 had higher probability of uveitis remission during the time of treatment on Adalimumab (median ±SE: 18 **±**1.1 *vs* 4 ±0.6 months, CI 95%: 15.6**-**27.5 *vs* 2.7-5.2, Mantel-Cox χ^2^10.12, p<0.002).

**Conclusions:**

Even if limited to a relatively small group, our study suggests a better efficacy of Adalimumab when used as *first* anti-TNFα treatment in childhood chronic uveitis.

## Background

Non-infectious, chronic uveitis in childhood is a relatively uncommon, but serious disease, with the potential for significant long-term complications and possible blindness [1-3]. To date, nonetheless the fare cumulative data, there is a compelling body of evidence that biologic modifier therapies are available treatment strategies for sight-threatening uveitis in children [4-16]. However, the lack of evidence from head-to-head randomized controlled studies limits our understanding of when to commence therapy, which agent to choose and how long to continue treatment, and, in case of failure, if switching to another anti-TNF-α strategy might be eventually an option. Currently, only an evidence level of III supports the treatment with immunosuppressive/biological modifiers drugs: expert opinion, clinical experience or descriptive studies.

We recently showed, in a multicenter, comparative prospective case series (evidence level IIb) the superior efficacy of Adalimumab compared to Infliximab in maintaining long-lasting remission in immunosuppressive-refractory childhood chronic uveitis [17]. Moreover, we reported a previous evidence of the loss of efficacy of Infliximab in long-term follow-up [18].

Nonetheless, the clinical question of which anti-TNF-α blocker seems more suitable for starting treatment in childhood auto-immune uveitis is still unsolved and debating.

In order to address this point, the purpose of this study was to compare the efficacy of Adalimumab, when used as first anti-TNF-α therapy, versus Adalimumab, used after the failure of a previous anti-TNFα (Infliximab), in childhood non-infectious chronic uveitis.

## Methods

### Study design

Prospective, comparative case series open-label study of pediatric patients with refractory uveitis treated with Adalimumab, for at least one year period at three tertiary pediatric rheumatology centers in Italy: Anna Meyer Children’s Hospital, Florence; Institute of Child Health IRCCS Burlo Garofolo, Trieste and Paediatric Clinic, University of Brescia.

### Inclusion criteria

To be considered eligible for this study, patients were required to have disease onset prior to 16 years, vision threatening non-infectious uveitis that was refractory to therapy with systemic corticosteroids and at least one other immunosuppressive medication, or to be intolerant to such therapy. ‘Refractory’ was considered as persistently active uveitis for at least 3 months despite systemic cortico-steroids and immunosuppressive treatment (Methotrexate [MTX] and/or Cyclosporin A [CSA]).

### Study and treatment protocol

At the time of enrolment, medical history and complete rheumatologic and ophthalmologic examinations were performed in addition to a tuberculin purified protein derivative skin test and a chest radiograph.

Due to the refractory course of uveitis to previous DMARD treatment, after stopping the previous immunosuppressive therapy (except corticosteroids), eligible children were consecutively enrolled in 2 groups, receiving Adalimumab at the dose of 24 mg/sq.mt, subcutaneously every 2 weeks, for at least 1 year:

• Group 1 received Adalimumab, as first anti-TNF-α choice, naïve from any anti-TNFα treatment;

• Group 2 received Adalimumab, as second anti-TNF-α drug, after a previous treatment course with Infliximab. In this group, when Infliximab, administered for at least 1 year at 5–10 mg/kg weeks at 0, 2, 6 and then every 6–8 weeks, lost efficacy due to persistent/refractory uveitis, children were then switched to Adalimumab. Of note, during Infliximab treatment, children had been receiving MTX treatment at very low dosages (5–7.5 mg/week) in order to prevent the formation of anti-infliximab autoantibodies.

At enrolment, steroid therapy was maintained at stable dose (prednisone 1–2 mg/kg/day) for at least 6 weeks, and then tapered once remission was achieved with regard to uveitis activity.

The choice of timing of Adalimumab as TNF-α inhibitor was an opinion-based decision of the treating ophthalmologist and rheumatologist in collaboration and on the basis on drug availability at their own center at the date of starting the anti- TNF-α therapy.

Every 30–45 days, children received a routine assessment, including a general physical examination, laboratory evaluation with renal and liver function tests, complete blood count and inflammation parameters and a complete ophthalmologic evaluation, including best corrected visual acuity (BCVA) on Snellen eye charts and slit lamp examination, that was performed at study enrolment and according to the degree of activity thereafter. Once uveitis achieved remission, children underwent an ophthalmologic evaluation at each assessment or otherwise on clinical demand as needed. In both groups, therapy was withdrawn if major side effects/complications due to the treatment increased and/or lack of efficacy appeared.

The exact same protocol was applied in the three centers. Each local ethic committee gave their approval. Parents or guardians gave their informed consent.

### Patients

All of the patients in this series were recruited from the Pediatric Rheumatology Units in Florence, Brescia, and Trieste from June 2007 to November 2010.

During the same period of the study, our centers were following a total of 188 pediatric patients with chronic uveitis (127 females, 61 males, median age 6 years, range 3–18 years); 119 were associated with juvenile idiopathic arthritis (JIA), 10 with Behçet’s disease, 1 with early-onset sarcoidosis, and 9 with other connective tissue diseases (systemic lupus erythematosus or mixed connective tissue disease), while the other 49 had idiopathic uveitis.

Twenty-six children (14 females, 12 males, median age 8.6, range 5.2-13.8 years) resulted in being eligible for the study and were enrolled: 17 were recruited in Florence, 6 in Trieste, 3 in Brescia. In 20 of 26 children, uveitis was associated with an underlying autoimmune disease: 17 JIA (9 oligoarticular, 5 extended oligoarticular, 3 RF-negative polyarticular), 1 early-onset sarcoidosis, 2 Behçet’s disease. The other 6 children had idiopathic uveitis. Among 9/20 patients with secondary uveitis, at enrolment, the associated underlying disease was active despite concomitant medications, while the remaining 11 patients were in remission on therapy with regard the associated disease, but not to uveitis. Before Adalimumab treatment, all children had presented active uveitis: 33/52 involved eyes, despite treatment with MTX at the dosage of 15 mg/m2/weekly (n = 17), CSA at the dosage of 3 mg/kg/day (n = 6) and the combined administration of MTX and CSA (n = 3). Due to active uveitis, along with topical steroids during the acute phase, all of the children were also receiving oral prednisone (1–2 mg/kg/day), at stable dose for at least 6 weeks (range 43–56 days).

### Main outcome measures

In order to assess long-lasting effect on maintaining remission, the primary outcome was to assess, once remission was achieved, the time of a first relapse during treatment. In addition, secondary outcomes were to compare, once anti-TNF-α treatment was started, time to uveitis remission, time to steroid discontinuation, and the number of uveitis relapses. According to the Standardization of Uveitis Nomenclature Working Group grading schemes, uveitis activity, as grade ≥ 1+, was defined improved when decreased by 2 steps in the level of inflammation or decreased to grade 0 [19]. As previously reported, “improved” visual acuity, converted into a logMAR format, was defined as a doubling, “worsened” as a halving of the visual angle in at least 1 eye [20].

### Statistical analysis

Mann–Whitney U test, Wilcoxon’s signed rank test for paired samples, chi-square tests, McNemar test, and Fisher’s exact test, when appropriate, were used to compare data. Two-tailed *P* values were employed. An a priori power analysis was completed using G Power program [21]. Considering current data of refractory uveitis in children, a large expected difference was estimated for the sample: the effect size F = 0.40, as per Cohen [22]. In addition, power was set at 0.95, meaning there would be a 95% probability of reaching statistical significance if the obtained differences were truly present in the population. Results from the power analysis showed that 28 participants, 14 for each arm, in all groups combined would be required. The following data, entered into a customized uveitis database, were considered as variables for correlations and as covariates for the survivage at the initiation of/age at the initiation of anti-TNFα therapy, gender, associated autoimmune disease, disease duration, age at uveitis onset, uveitis duration, active uveitis duration, time interval between the uveitis onset and the initiation of anti-TNFα therapy, concomitant medications, previous cumulative corticosteroid dose and its duration, previous disease modifying anti-rheumatic drug treatment duration, number of previous flares, number of patients with eye complications due to chronic uveitis (including glaucoma, synechiae, band keratopathy, cystoid macular edema, vitreitis, and cataract), and follow-up time. Pearson’s and Spearman’s correlation tests were used to determine correlation coefficients for different variables. In order to identify predictors of outcome, Cox regression model and Kaplan-Meier curves were constructed, each one at the mean of the covariates reported above. All analyses were performed with the SPSS package for Windows, version 13.0.

## Results

Fourteen children (9 females, 5 males), 10 affected by JIA, 3 by idiopathic uveitis, and 1 by Behçet’s disease, were recruited in Group 1, thus receiving Adalimumab as first anti-TNF-α drug. Twelve children (7 females, 5 males), of whom 7 were affected by JIA, 3 by idiopathic uveitis, 1 by early-onset sarcoidosis, and 1 by Behçet’s disease, were enrolled in Group 2, who received Adalimumab as second anti-TNF-α drug.

The total median length of uveitis duration before Adalimumab treatment was significantly higher in Group 2 than in Group 1: 28 months, range 22–34, *vs* 16 months, range 12–22 (p = 0.001). Demographic information and other reported variables in the statistical analysis section, acting as covariates, did not differ in the two groups. During the time of treatment, Adalimumab was not able to control eye inflammation during the first year of treatment in one JIA child, belonging to Group 1; therefore, she never achieved remission and was considered “non-responder”. She therefore resulted in being eligible for the inclusion criteria (refractory uveitis), but not for our primary outcome measure (absence or recurrence of uveitis), and therefore was excluded from the long-term survival analysis.

Cox-regression analysis, at mean of the above mentioned covariates, including the total length of follow-up time, did not show statistical significant differences between the two groups with regard to time to achieve remission, even though a longer, but not significant, time to achieve remission was observed for Group 2: median period (range) 16 weeks (range 12–18) *vs* 12 weeks (range 8–16).

**Figure 1:**
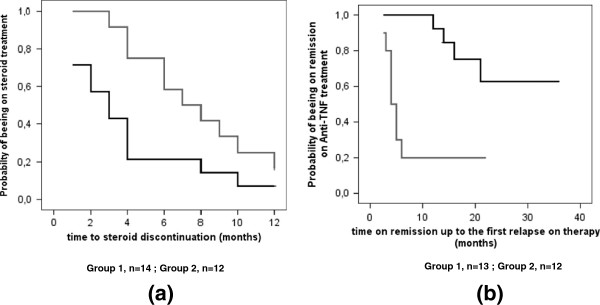
**Time to steroid discontinuation and time on remission up to the first relapse on Adalimumab. a**. Survival curves of time to steroid discontinuation (months) for the Group 1 (***black curve***), receiving Adalimumab, as first anti-TNFα therapy, and the Group 2 (***grey curve***), receiving Adalimumab as second anti-TNFα therapy. On the y-axis, the probability of patient being on steroid treatment is shown. (log-rank, Mantel-Cox χ^2^ 4.12, p < 0.004). **b**. Survival curves up to the first uveitis relapse on therapy after achieving remission (months) for Group 1 (***black curve***), receiving Adalimumab as first anti-TNFα therapy, and Group 2 (*grey curve*), receiving Adalimumab as second anti-TNFα therapy. On the y-axis, the probability of patient being on remission on anti-TNF-α therapy is shown (log-rank, Mantel-Cox χ^2^ 10.12, p < 0.002).

Seventeen children (12 in Group 1 and 5 in Group 2) were able to stop steroid administration during the first 6 months from the start of Adalimumab, and all responders discontinued steroid before 1 year of treatment. However, Cox-regression showed that patients in Group 2 needed a longer time to discontinuation of steroids (median ± SE: 7 ±1.7 *vs* 3 ±0.9 months, CI 95%: 3.6-10.4 vs 1.1-4.8, p < 0.001) and a lower probability to steroid discontinuation during the first 12 months of treatment (log-rank, Mantel-Cox χ^2^ 4.12, p < 0.004) (Figure 1a).

With regard to our primary outcome measure, at the mean of the above mentioned covariates, including the total length of follow-up time of the 2 groups, Cox regression analysis showed that, during the time of treatment on Adalimumab, Group 1, compared to Group 2, had higher probability of uveitis remission, considered as time to first flare, (log-rank, Mantel-Cox χ^2^ 10.12, p < 0.002) and longer time on remission on treatment: median ± SE: 18 ±1.1 vs 4 ±0.6 months, CI 95% 15.6-27.5 vs 2.7-5.2, p < 0.001) (Figure 1b).

During the first year of treatment, no relapse of uveitis occurred in all 13 responders on Group 1, whilst, among 12 children in group 2, just 2 children with JIA were still on remission, while the remaining 10 children experienced a median number of relapses of 2 (range 1–5).

**Figure 2:**
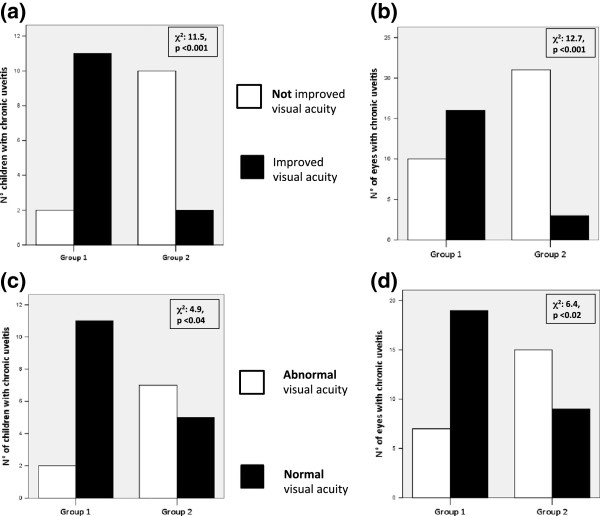
**Improved and normal visual acuity on Adalimumab. a-b**. Number of children (**a**) as well the number of eyes (**b**) with improved (***black bar***) and not improved (***white bar***) visual acuity at 1 year of treatment in Group 1, receiving Adalimumab as first anti-TNFα therapy, and Group 2, receiving Adalimumab as second anti-TNFα therapy. (χ^2^: 11.5, p < 0.001; and χ^2^: 12.7, p < 0.001, respectively). **c-d**. Number of children (**c**) as well number of eyes (**d**) with normal (***black bar***) and abnormal (*white bar*) visual acuity at 1 year of treatment in Group 1, receiving Adalimumab as first anti-TNFα therapy, and in Group 2, receiving Adalimumab as second anti-TNFα therapy (χ^2^: 4.9, p < 0.04; and χ^2^: 6.4, p < 0.02, respectively).

After the 1-year follow-up visit, among responders, 11 (84.5%) of 13 children in Group 1 and 2 (16.5%) of 12 children in Group 2 met the criteria for improved visual acuity (χ^2^: 11.5, p < 0.001), corresponding to 16 (61.5%) of 26 eyes and 3 (12.5%) of 24 eyes, respectively (χ^2^: 12.7, p < 0.001) (Figure 2a-b).

At the 1-year follow-up, the number of patients as well as the number of eyes within a “normal visual acuity” was significantly higher than before treatment for Group 1 (11/13 patients *vs* 4/13 patients, p <0.008; and 19/26 eyes *vs* 7/26 eyes, p *<*0.001), whilst we did not observe significant differences with regard to Group 2 (5/12 patients *vs* 4/12 patients; and 9/24 eyes *vs* 8/24 eyes).

At 1 year of treatment, we detected significant differences between the two groups with regard to the number of children and number of eyes within a “normal visual acuity” (χ^2^: 4.9, p < 0.04; and χ^2^: 6.4, p < 0.02, respectively) (Figure 2c-d).

In addition, we performed a sub-group analysis limited just to children with JIA: 9 belonging to the Group 1, and 7 to the Group 2. With regard to our primary and secondary outcomes, we obtained similar statistical results. Cox regression analysis showed that Group 1 had a higher probability of uveitis remission compared to Group 2 (log-rank, Mantel-Cox χ^2^ 12.83, p < 0.002), and longer time on remission on treatment: median ± SE: 19 ±0.9 vs 6 ±1.2 months, CI 95% 16.5-28.6 vs 3.6-6.4, p < 0.001). We did not observe any statistical differences between the two groups with regard to the time to uveitis remission. Instead, children in Group 1 have been able to discontinue systemic steroid administration before than children in Group 2 (median ± SE: 2 ±0.3 *vs* 6 ±0.7 months, CI 95%: 1.1-3.8 vs 3.6-8.4, p < 0.001), and had higher probability to stop steroid during the first 12 months (log-rank, Mantel-Cox χ^2^ 5.21, p < 0.003).

At 1-year follow-up visit, 8 (88.8%) of 9 children in Group 1 and 2 (28.5%) of 7 children in Group 2 met the criteria for improved visual acuity (χ^2^: 6.11, p < 0.03), corresponding to 11 (61.1%) of 18 eyes and 3 (21.4%) of 14 eyes, respectively (χ^2^: 5.03, p < 0.03).

None of the patients were amblyopic and refractive errors were corrected by means of glasses or contact lenses. All recorded variations in best-corrected visual acuity were therefore related to disease activity and no clearance of media was recorded.

## Discussion

Even if limited to a relatively small group, this comparative cohort-study suggests a better efficacy of Adalimumab when used as first anti-TNF-α treatment in chronic childhood uveitis, with regard to time of first flare, once remission has been achieved.

To our knowledge, RCTs on this topic have not yet been published to date, and our study represents the first prospective cohort comparative study assessing differences in timing use of anti-TNF-α treatment for childhood chronic refractory uveitis. Based on these data, if our results will be duplicated in a larger and homogenous cohort, the evidence of this different timing in using anti-TNF-α therapy will reach a level of evidence of IIb, better than the actual knowledge (level of evidence of III).

Recently published case series suggested that in case of refractory uveitis with lack of initial clinical response to one anti-TNF-α agent, switching to another one could achieve control of intraocular inflammation [23]. Accordingly, Biester et al. also reported favorable results with Adalimumab when Infliximab or Etanercept were not effective or not tolerated, but also good effects with Etanercept or Infliximab in case of lack of response to Adalimumab [24]. Conversely, our study does not seem to confirm these findings. However, the former studies reported their mono-center experience by a retrospective analysis on smaller, not comparative, case series; we addressed this topic by a prospective comparative analysis of 26 children from different Italian centers. The different approach could explain at least in part these differences.

Of note, before drawing firm conclusions from our results, some caveats have to be discussed and considered. Of course, the small sample size due to the rarity of the disease limits our study results. The heterogeneity of the sample cohort, with regard to the underlying disease as well as to the inherent selection bias of 3 tertiary referral centers, might also affect the results of our study, overweighting the obtained data. However, our study population resulted homogenous for the primary outcome study (childhood refractory chronic uveitis), and, due to the small number of potential eligible subjects, a more conservative inclusion criteria of population would hamper the a-priori power analysis, thus stronger affecting the results. The longer follow-up period, thus the longer disease duration, in the Group 2 could have been inferring the data, resulting in a non-homogenous comparison. In addition, children in Group 2, receiving much longer treatment, not controlling the disease, might have been also acquired an irreversible damage, losing the chance to improve, regardless the administered treatment. Hence failure to respond to Adalimumab would not be surprising. We thought to minimize this effect bias considering the length of follow-up as a covariate for the survival curves, therefore overweighting its potential effect size.

Conversely what we previously reported [17], the present study seems to show that Adalimumab looses the capacity to maintain longer remission period if used as second anti-TNFα treatment in childhood chronic uveitis, refractory to a previous anti-TNF treatment (Infliximab). We are not able to explain this biological phenomenon and it is also likely that this is not specific for Adalimumab neither for childhood uveitis, since other anti-TNF agents in different diseases (i.e. Rheumatoid Arthritis) can do the same. Even though our study design is not able to address this point, we can hypothesize that, during the biological modifiers administration, the immune system, with their ongoing use, learns to drive on alternative pathways, bypassing the TNFα blockade. In this clinical setting, switching to another class of biological modifiers drugs, such as Abatacept, seems a reasonable strategy rather than maintaining an anti TNF-α option. Indeed, in our clinical practice of childhood chronic uveitis, when a first anti-TNF-α treatment fails, we switch to the co-stimulatory blocker [25,26].

## Conclusions

Clinical practice guidelines and consensus statements on the criteria of introduction, duration of treatment and cessation of TNF antagonists, including safety issues, are strongly advocated and a constant revision as data from longer periods of patient exposure accumulate is warranted. Facing children with autoimmune chronic uveitis, refractory to steroids as well as to conventional immunosuppressant therapy, prone to a clinical dilemma: which anti-TNF-α strategy should we prescribe for that child? Even if not coming from an evidence level of I or II, it is a diffuse experience that Etanercept is not suggested as a first choice treatment in refractory childhood auto-immune chronic uveitis [3,27]. The present comparative prospective study, along with the former one [17], might suggest clinicians to consider Adalimumab as potential starting option in case of childhood chronic refractory uveitis.

Further studies in a larger cohort, in a prospective fashion, preferably by a randomized clinical trial, focused on one disease entity with a sufficient sample size, seem to be advocated to address this point.

### Key messages

Adalimumab, when used as first anti-TNF-α treatment in chronic, refractory childhood uveitis, shows a better efficacy than used as second line anti-TNF-α agent.

In clinical practice, Adalimumab might be the first starting option in case of refractory childhood uveitis.

## Competing interests

The authors declare no conflicts of interest.

## Authors’ contributions

GS, AT, MC contributed to the design of the study, performed data and study sample collection, analysis and interpretation. IP performed data and study sample collection. RC, CdeL, and FP performed all the ophthalmologic follow-up and carried all the related data. GS carried out statistical analysis and wrote the manuscript. AT, MC, LL and RC helped in revision and suggestions. All authors read and approved the final manuscript.
